# Dr. Young, on Consumptive Diseases

**Published:** 1817-04

**Authors:** 


### *Dr.* YOUNG, *on Consumptive Diseases.*

(Continued from our last Number, p. 237.)

CHAPTER 5. *Causes of Consumption.* Of the essential cause of consumption little is known. That it consists in no peculiar acrimony of the sytem is proved by cases in which the symptoms have subsided on the removal of a foreign substance by which they had been induced: nor is it always attributable to mechanical irritation. The disease seems to be mostly dependent on a peculiarity of constitution, which operates by the production of tubercles in the pulmonary structure; and probably the causes which are thought to excite the malady, may, in general, not exist previously to the formation of these morbid substances. The original causes of this constitutional peculiarity are nearly the same with those of scrofula, as debilitating excesses, want of proper aliment. The remains of other diseases, or the mischievous influence of the treatment which they require, and especially removal from a warm to a cold and variable climate, often act as the remote causes of consumption. To this catalogue we beg leave, for the sake of our fair country-women, on whose

---

minds the dangers with which it is pregnant cannot be too deeply impressed, to add another cause of frequent occurrence: A sudden transition from the heated atmosphere of the crowded theatre or ball-room, to the chilling midnight air of a northern winter; and that too by delicate females, arrayed in the spare and flimsy costume of the fashionable world. To our vulgar admonitions on this subject, experience has long taught us that the female ear is utterly insensible. But when we see youth, and beauty, and intellect, thoughtlessly sacrificed at the shrine of the devouring Moloch, Fashion, we should think it a criminal negligence to be silent. It is surely the duty of medical practitioners to point out, to their female patients, the danger of such imprudent and indecorous scantiness of covering, and earnestly to protest, both in public and private, against it. We shall terminate this digression by observing, that if women could but know the feelings of that part of the male sex, whose admiration and respect it is their interest only to conciliate, on this subject, such knowledge would shew them the *impolicy*, far more effectually than we can hope to convince them of the *dangers*, of the present frigid system of fashionable decoration.

The consumptive constitution is often hereditary; and its existence signalized by a set of physical characters, with which the reader must be sufficiently familiar. Long dark eye-lashes and a large pupil are regarded as indicative of the phthisical habit. Our experience leads us to believe, that these appearances will be most commonly, if not exclusively, observed in those cases of consumption which originate from, or are connected with, a morbid state of the mesenteric glands. They are certainly often very strongly marked in subjects predisposed to scrofula. The hereditary predisposition is sometimes so decided as to induce consumption without any external exciting cause, for ulcers and tubercles have been found in the fœtal lungs. But generally the disease is referable to some pulmonary irritation, giving rise to tubercular deposition, or exciting inflammatory action in the parts which already constitute the seat of such depositions, particularly inhalation of the dust of hard substances. We believe with Dr. Young, that the smoke of towns is less injurious in this respect than is commonly imagined. We too have seen symptoms of pulmonary disease, originally developed in the country air, removed by a month's immersion in the loaded atmosphere of the metropolis, and without the aid of any other remedy. How far the accusations of foreigners may be correctly founded, with regard to the noxious influence of coal, in a state of combustion, on the respiratory organs, we presume not to determine; but certain it is, that a native of the continent, unaccustomed to respire the effluvia of burning coal, experiences, on first approaching an English fire-side, a very distressful sense of pulmonary irritation. To the general opinion, that consumption most frequently occurs between the ages of 18 and 35, Dr. Young does not assent. If the evidence of registers be consulted, it will be found more destructive above, than below, the latter period; and very rare below the twelfth year. The mesenteric decline mostly affects children. Nasal hæmorrhage has been known to prevent consumption. Nutritious diet with exercise is its most effectual prophylactic. Butchers rarely suffer from it. Amenorrhœa, though regarded by French physicians as the cause, is probably rather a consequence, of it. The disease has usually its progress suspended, and sometimes permanently arrested, by the supervention of pregnancy. The same effect may result from cutaneous eruptions, which seem to operate on the principle of counter-irritation.

Much difference of opinion has prevailed as to the possibility of consumption being propagated by contagion. We agree with Dr. Young, that no satisfactory evidence of its contagious nature has yet been adduced. At the same time, we by no means think, that even a healthy individual could long respire with impunity the effluvia issuing from the lungs of another in a state of suppuration. Whether, however, such effluvia would merely excite a general derangement of the system, or operate specifically on the pulmonary organs, and thus induce an analogous lesion of structure, is a question hitherto undecided, and perhaps likely always to remain so. But, at all events, it would be highly impolitic to expose any one, and more especially a person of weakly or consumptive habit, to a source of danger, which, in general, may be readily obviated. That two or three individuals of a family are successively, and within a very short period, destroyed by pulmonary consumption, is a fact which has frequently occurred, and been often adduced by the partizans of the contagious nature of phthisis, in confirmation of their doctrines; but such fact should, by no means, be regarded as decisive of the question. Predisposition to phthisis, when strongly marked and hereditary, frequently pervades all the members of a family. The first of them, in whom the dormant spark is aroused into action, dies after a struggle, protracted perhaps during many months. Some near relative of the deceased, who has nursed him in his confinement, and felt anxiously interested in his fate, next droops, and falls a victim to the unsparing malady. Hence arises the inference, that the last unfortunate event must necessarily have been the consequence of contagion. But in these calculations ought it to be overlooked, that the second victim of the disease shared with the other, and probably in a very conspicuous degree, the constitutional peculiarities of his race; that the predisposition to pulmonary disease, which might otherwise have lain dormant for a time, was perhaps excited by the want of accustomed exercise, by nightly wakefulness and exposure, by anxiety; in fact, by the conjoint operation of all those physical and moral causes, to which confinement in the sick room of a relative is calculated to give existence and energy, and which, by depressing the vigour of the system, will explain the occurrence of phthisis, independently of all inhalation of noxious effluvia? * We have been tempted to submit these views to the consideration of the reader, in that free spirit of discussion which, while it bends not in servile acquiescence to opinions, merely because they are generally received or powerfully sanctioned, is far from aiming to establish others, for the sake of novelty or disputation. Truth is our exclusive object; and we again allow, that, in the present undecided state of this important question, it would be unjustifiable needlessly to "expose any person, who appeared to have the slightest predisposition to the disease (or otherwise), to any intimate communication with a consumptive patient." Abscesses of the liver, gaining adhesion to the diaphragm, sometimes burst into the lungs, and induce all the phænomena of pulmonary consumption. In such case, the expectoration contains a mixture of bile; but the disease is not always incurable. A case is mentioned by Dr. Young, in his *Medical Literature,* "which was rendered inevitably fatal by the total

---

* We remember, some years since, to have seen a stout, athletic man, of the middle age, not evincing the slightest predisposition, hereditary or acquired, to pulmonary disease, attacked with the incipient symptoms of *bronchial* phthisis, in a very decided form, during a long and close attendance on his wife, while struggling against incurable *tubercular* consumption; but we were inclined to attribute the origin of this affection to loss of rest, frequent exposure to the night-air, and the influence of other physical and moral impressions connected with his situation. He was permanently restored to health by change of air, counter-irritation, and dietetic restrictions.—Rev.

obstruction of the gall-duct, into which a large hydatid had descended from the abscess, so that the whole of the bile was obliged to find a passage through the lungs." Hepatic affections also not unfrequently operate as the exciting causes of pulmonary consumption.

CHAPTER 6. *Treatment of Consumptive Diseases.* They who, year after year, have been accustomed to witness the almost invariable failure of every attempt to arrest permanently the progress of consumption, will turn to this chapter with no feelings of sanguine expectation, and will probably quit it without disappointment; for, although destitute of any very novel feature, it contains many judicious remarks, evidently the results of diligent observation and long experience: but, perhaps, they are not sufficient in number or originality to remunerate us for a rigorous analysis of the whole chapter. Symptomatic *hectic fever* is only to be radically cured by removal of the primary disease. Digitalis is not to be depended on as an antihectic, and is useless in simple hectic affections. Anodynes, camphor, ipecacuan, may be employed to allay the febrile irritation. The sulphuric acid is the most powerful remedy for night sweats. Ipecacuan, alone or mixed with chalk, log-wood, or opium, may be often successfully used against the diarrhœa of hectic; but if, from its obstinacy, incipient intestinal ulceration is suspected, calomel, combined with opium or otherwise, should be tried. This remedy will often be found serviceable in declines, especially "such as depend on intestinal or visceral derangements." Steel, myrrh, and vegetable bitters, are the best tonics.

In *mesenteric decline,* calomel in small doses constitutes the chief remedy. It may be combined with hemlock, and aided by occasional laxatives, even though diarrhœa exist. Sometimes the intestinal discharge may require palliation by opium, ipecacuan, and astringents. If the calomel disorder the bowels, mercurial friction may be substituted for it, with care to discontinue it as soon as the system exhibits signs of the mercurial action. Steel, and vegetable tonics, particularly infusion of Angustura bark, will be found excellent remedies in the later periods of the disease; which, by judicious application of these means, may frequently be conducted to a fortunate issue.

Dr. Young next proceeds to consider, separately, the operation of the principal remedies which seem to have been most successfully employed in *pulmonary consumption,*

and to deduce, from the results of experience, the circumstances under which each of them is respectively applicable. These remedies he arranges in the following order: *Bleeding*, general and local; *cathartics*, as neutral salts, calomel, and sulphur; *emetics* — ipecacuan, tartar emetic, the sulphates of zinc and copper; *sorbefacients* — digitalis, mercury, alkalies; *epispastics* — blisters, issues, caustics, and setons; *sudorifics* — antimony, Dover's powder, sarsaparilla; *expectorants* — gum ammoniac, squill, polygala; *demulcents* — oils and gums; *narcotics* — opium and hemlock; *suppuratories*—balsams and balsamic vapours; *astringents* — mineral and vegetable acids, catechu and kino; *tonics*—steel, myrrh, bark, Angustura, lichen; *diet; exercise;* and *climate*.

In speaking of *emetics*, Dr. Young extols the employment of ipecacuan in terms which, we lament to say, our own experience of its powers in pulmonary consumption, will by no means authorize. He considers this root preferable to antimony or the sulfates of copper and zinc, as an emetic, "from its singular power of subduing hæmorrhages of all kinds, and of restraining some other excessive discharges:" indeed, he is disposed to regard it as "a medicine little, if at all, inferior in importance, to cinchona or mercury." He depends upon it alike in hæmoptysis, for which he thinks it a more safe and permanent remedy than acetate of lead, and in cases of seemingly purulent expectoration which he has sometimes seen removed by its continued use; but, more commonly, the secretion has been only, for a time, improved in quality and diminished in quantity, by it. However successfully ipecacuan may be administered in the *early stages* of consumption, when abstinence may operate beneficially with other means, in reducing the quantity, and moderating the impetus of the circulating fluids, we find that it can rarely be employed with advantage, or even with impunity, in *confirmed cases*, where the system is sinking under a profuse purulent secretion: for, even in minute doses, and when combined with opium and aromatics, it is liable to produce distressing nausea, depression, and loss of appetite, by which the already existing weakness and exhaustion are signally aggravated. This effect is, if we mistake not, most commonly and conspicuously exhibited where the pulmonary affection is complicated with a morbid state of the mesentery or intestinal canal.

Sufficient stress has, we think, scarcely been laid by Dr. Young on the employment of *large issues*. In those species of phthisis, which Dr. Duncan designates by the terms *apostematous* and *catarrhal*, as resulting from unsubdued pneumonia, or a morbid condition of the bronchial membrane, we have seen the establishment of purulent secretion from an extensive surface most decidedly beneficial. A small issue will do nothing, but needlessly irritate the patient. To employ the language of a facetious pulmonic, some years since under our care, the sternal region should be "converted into a pea garden." We have rarely seen good result from the action of blisters, in these modifications of phthisis; but frequently mischief, in the *tubercular* species, by increase of the general irritation and debility. The ointment of tartrite of antimony is more favourably spoken of by Dr. Young than we, from unprejudiced and tolerably extensive observation of its effects in the treatment of consumptive diseases, consider that it deserves. It is a fact little suspected by the generality of practitioners, that sudorifics, as the compound powder of ipecacuan, given in the evening, is sometimes the most effectual remedy for repressing the *night sweats* of hectic. The assertions of a man like Dr. Young can little require the aid of our testimony, or we might adduce it as to the correctness of this valuable observation.

On the subject of *astringents*, Dr. Young principally restricts his observations to sulfuric acid. Of the employment of the *acetic*, as described in the fifth volume of the *Medical Transactions*, he probably was not apprized, when writing this work. During the last three months, we have tried this remedy in one case of confirmed and three of incipient phthisis. In the former, which was evidently connected with diseased mesentery, it aggravated the intestinal pain and irritation, without at all relieving the pulmonary symptoms. Assisted by local bleeding, abstinence, and *pilula hydrargyri*, it was productive of the most signal and unexpected benefit, in one of the incipient cases: in the second, it evinced no sensible effect. The issue of the third, and perhaps most unpromising case, is yet undecided; but all the more formidable symptoms have already yielded under the employment of the acetic acid, to which was added the *tinctura lyttæ* in such doses as produced considerable irritation of the urinary organs. Some part of the integuments of the chest has also, in this instance, been kept constantly irritated by the antimonial ointment. At all events, the Moorish plan of treatment is certainly entitled to a full and patient trial in phthisical diseases. We venture, however, to predict, that it will not prove applicable to cases where the pulmonary malady is complicated with intestinal affections, and particularly a morbid condition of the mesenteric glands.*

Dr. Young, in his observations on the *dietetic plan*, best suited to consumptive patients, declaims strongly against the liberal allowance of animal substances and wine. But if he mean rigorously to exclude these articles from *every* stage and modification of phthisis, not even the dread of being stigmatized as "theorists," or having our judgement impeached as "practitioners," will induce us to assent to his prescription. No man can more justly appreciate, than ourselves, the advantages of rigid abstinence in cases of incipient phthisis, or more strongly recommend its adoption. But we have long been convinced, and every day's experience serves only to strengthen this conviction, that, in confirmed phthisis, where purulent expectoration is decidedly established, and particularly in the latter stages of *mesentero-pulmonary consumption*, a moderate, and even under some circumstances liberal, allowance of solid animal food and generous wine, will signally contribute to alleviate the sufferings, and protract the existence of the patient. Nay, we have seen, and certainly not at first without dread of self delusion, and feelings of astonishment, the cough, the hectic fever, and the night sweats subside, and the expectorated pus assume a more healthy appearance, exactly in proportion to the increased quantity of this commonly-forbidden aliment which could be comfortably taken and retained by the patient's stomach. And how much of such substantial nutriment will, under these circumstances, be consumed by the pulmonic, not only without inconvenience, but with obvious advantage, none, except they who have had frequent opportunities of observing for themselves, would be disposed to credit. On the subject of *climate*, we are referred to the succeeding chapter.

CHAPTER 7. *An Essay on the Medical Effects of Climates.* This Essay is reprinted from the author's work on *Medical Literature*, a publication which, from its well-merited reputation, and from the circumstance of its being nearly out of print, must be familiarly known or

---

* *See* the papers xviii and xxix, in the fifth volume of the *Medical Transactions of the College of Physicians*; or the review of them, in the tenth number of the *Med. Chir. Journ. and Review*. The pills, of Moorish prescription, we have not thought it necessary to employ.—REV.

possessed, by every respectable practitioner. On this account, we shall pass more rapidly over the present chapter than we otherwise should have done. In his researches on the temperature of various parts of this island, of Paris, Malta, Madeira, and Jamaica, Dr. Young has found, from cautious comparison of their respective meteorological registers, that none of these situations, North of Lisbon, except Penzance, possess any decided superiority over London, in the mildness of its winter. The temperature of Penzance is, in the coldest months, 4½° higher than that of the metropolis: while that of the warmest parts of Devonshire has only the superiority by about a degree and a half. Torquay, it is supposed, may be somewhat milder than this. It is singular that the cities of Edinburgh and Paris should possess, during the three coldest months, an equality of temperature; which temperature is about three degrees lower than that of the English capital. Hastings, and other sheltered situations of the Sussex and Hampshire coast, may be less exposed to the north and easterly winds, than other parts of our island. But, in proceeding up the channel, the influence of the mild gales of the Atlantic is proportionately less felt; and the south-west winds, considerably cooled in their passage across the continent, are encountered. Both Malta and Madeira, Dr. Young observes, present numerically, a mean temperature for the winter months, as favourable for an invalid as can possibly be desired."

With respect to equability of temperature, another very important consideration in pulmonary diseases, the air of Devonshire does not seem superior to that of London; but here, again, Penzance retains its advantage. The climate of Lisbon is less variable than that of any part of Britain; and Madeira, in this view, offers a still preferable situation. In a sea voyage to a warmer climate, however, the greatest equability of temperature may be obtained; and, in addition to the benefits of sailing, the pulmonic will thus avoid the fatigues attendant on travelling, and the danger of damp beds.

Consumption is more frequent in cold than in hot climates, but not in precise ratio to the depression of their mean temperature. And, in this respect, Dr. Young proceeds to compare the following situations with London, as a standard:

*The southern coasts of England.* Of their proportionate mortality from consumption, no sufficiently correct and extensive returns are to be obtained; but such statements, it is believed, would be highly favourable to their salubrity. Still, however, the advantages to be derived by a consumptive patient from residence in Devonshire or Cornwall, will not be equal to those which are presented by more remote situations, especially very small islands; as they, for equability of temperature, have a decided superiority to all others.

*The South of Europe.* In the Spanish Peninsula, consumption, though not common, is, by no means unknown; and the climate of Portugal has, in many instances, failed to arrest its progress. The mortality from the disease appears to be quite as great in the South of France as in London: not so with Italy, the climate of the southern parts of which approaches in mildness to that of the neighbouring islands.

*The islands of the Mediterranean.* Some of these, in point of temperature, probably possess great advantages over the surrounding continent. In the island of Malta, the thermometer rarely varies more than 6° during the whole day, or stands below 51°, even at mid-winter; while in Lisbon and Naples, at that season, ice and snow are sometimes seen, and the thermometrical difference between day and night often amounts to 20°.

*The island of Madeira* is perhaps a more pleasant residence than Malta, but seems to boast no decided superiority over it in regard to climate; and greater equability of temperature, it is supposed, may be found in some of the neighbouring islands. Yet, the beneficial effects of its climate in pulmonary diseases, have been established by long experience. Consumption there is comparatively rare. In the *West Indies,* the disease, like scrofula, rarely occurs; and very favourable reports of the influence of the climate have been given by several practitioners: and, if an inference may be drawn from the characters of a climate, as indicated by the thermometer, or by its effects on the human system, a residence in Bermudas, Jamaica, or some other of the West India islands, with the equable temperature of the sea air during the voyage, would probably afford to the migrating pulmonic every advantage which can be expected from climate alone.

But "in whatever situation the residence of an invalid may be fixed, it is of no small importance that the aspect and exposure of the house, which he occupies, should be selected with a view to the qualities of the climate he is desirous of obtaining. We have an illustration of the truth of this remark, in an observation recorded by Dr. Carrick, respecting the influenza of 1803. 'One of the most open and exposed of the buildings on Clifton Hill is

Richmond Terrace, which forms three sides of a parallelogram, fronting respectively the East, South, and West; on the East side, not one family, and scarcely an individual, escaped the complaint, while on the South side, a great majority, both of persons and families, in all other respects similarly circumstanced, escaped it entirely.' Such facts as these are among the few which afford solid ground for medical reasoning, and they deserve the more attention as they relate to circumstances of continual occurrence, and of perpetual influence on our health and comfort; and in proportion as both the medical and meteorological sciences become founded on a firmer basis, it cannot be doubted that their beneficial effects will be more and more experienced, as well in the preservation of health as in the treatment and cure of diseases."

The chapter is terminated by a *Table of the Annual Mortality of the different Counties of Great Britain, according to the Returns of* 1811. This we cannot condense, and have not room to insert.

BOOK II. *Medical History.*

This portion, by far the most considerable of the volume, contains a list of all the principal authors on consumption from Hippocrates to those of the present day, in the historical order, with an analysis of their respective opinions on its nature and treatment. The names of between three and four hundred writers are comprehended in the list. The whole reflects great honour on the learning and industry of Dr. Young. But, condensed as it is, we shall decline an attempt at analysis, which could prove neither creditable to ourselves nor profitable to the reader.

